# Ovarian Hyperstimulation Syndrome (OHSS): A Narrative Review and Legal Implications

**DOI:** 10.3390/jpm14090915

**Published:** 2024-08-28

**Authors:** Giuseppe Gullo, Gaspare Cucinella, Vukasin Stojanovic, Mirjana Stojkovic, Carmine Bruno, Adriana Vita Streva, Alessandra Lopez, Antonio Perino, Susanna Marinelli

**Affiliations:** 1 Department of Obstetrics and Gynecology, Villa Sofia Cervello Hospital, IVF Unit, University of Palermo, 90146 Palermo, Italy; gaspare.cucinella@unipa.it (G.C.); adriana.streva@gmail.com (A.V.S.); alessandralopez91@gmail.com (A.L.); antonio.perino@unipa.it (A.P.); 2 Emergency Medicine Center of Montenegro, Faculty of Medicine, University of Montenegro, 81000 Podgorica, Montenegro; vukasinstojanovic01@gmail.com; 3 Clinic of Endocrinology, Diabetes and Metabolic Disorders, University Clinical Center of Serbia, School of Medicine, University of Belgrade, 11000 Belgrade, Serbia; mirjanastojkovic@gmail.com; 4 Department of Medicine and Translational Surgery, Università Cattolica del Sacro Cuore, 00168 Rome, Italy; carmine.bruno@outlook.it; 5 Istituto Dermopatico dell’Immacolata (IDI IRCCS), 00167 Rome, Italy; 6 School of Law, Polytechnic University of Marche, 60121 Ancona, Italy; susanna.marinelli@tiscali.it

**Keywords:** ovarian hyperstimulation syndrome (OHSS), assisted reproductive technology (ART), gonadotropin-releasing hormone (GnRH) antagonist protocol, medicolegal viability

## Abstract

Background: Infertility is a highly meaningful issue with potentially life-changing consequences, and its incidence has been growing worldwide. Assisted reproductive technology (ART) has made giant strides in terms of treating many infertility conditions, despite the risk of developing ovarian hyperstimulation syndrome (OHSS), a potentially life-threatening complication. Methods: This narrative review draws upon scientific articles found in the PubMed database. The search spanned the 1990–2024 period. Search strings used included “OHSS” or “ovarian hyperstimulation” and “IVF” and “GnRH” and “hCG”; 1098 results were retrieved and were ultimately narrowed down to 111 suitable sources, i.e., relevant articles dealing with the condition’s underlying dynamics, management pathways, and evidence-based criteria and guidelines, crucial both from a clinical perspective and from the standpoint of medicolegal tenability. Results: The following features constitute OHSS risk factors: young age, low body weight, and polycystic ovarian syndrome (PCOS), among others. GnRH antagonist can substantially lower the risk of severe OHSS, compared to the long protocol with a gonadotropin-releasing hormone (GnRH) agonist. However, a mild or moderate form of OHSS is also possible if the antagonist protocol is used, especially when hCG is used for the final maturation of oocytes. For women at risk of OHSS, GnRH agonist trigger and the freeze-all strategy is advisable. OHSS is one of the most frequent complications, with a 30% rate in IVF cycles. Conclusion: Providing effective care for OHSS patients begins with early diagnosis, while also evaluating for comorbidities and complications. In addition to that, we should pay more attention to the psychological component of this complication and of infertility as a whole. Compliance with guidelines and evidence-based best practices is essential for medicolegal tenability.

## 1. Introduction

Nowadays, infertility is an extremely impactful issue with growing incidence worldwide, for which assisted reproductive technology (ART) has been playing an increasingly relevant role. Nonetheless, although ART is generally considered a safe option to achieve conception, it could still entail risks, such as the potentially life-threatening complication known as ovarian hyperstimulation syndrome (OHSS) [1,2]. OHSS is an important iatrogenic complication caused by ovarian stimulation which is associated with increased luteinizing hormone (LH), follicle-stimulating hormone (FSH), human chorionic gonadotropin (hCG), and estradiol (E2) levels [3]. OHSS etiological factors have been reported to include increased secretion or exudation of protein-rich fluid from ovaries or peritoneal surfaces, increased follicular fluid levels of prorenin and renin, and capillary permeability alterations linked to angiotensin mediation [4]. Some studies involving patients with an active mutation of the FSH receptor, or LH/hCG receptors resulting in spontaneous OHSS, have emphasized the role of gonadotropins as initiators of this condition [5,6]. OHSS has mild, moderate, severe, and critical forms, and the mild form is the most frequent [7]. This review article aims to shed light on the currently available research findings laying out OHSS’s underlying dynamics, pathophysiology, diagnostic, and therapeutic pathways, as well as evidence-based management strategies overall. A close focus on such complexities, and the implications of them, can decisively contribute to the clinical as well as the medicolegal viability of any approach for the sake of patient care. An overview of the forms of OHSS, with the frequency of occurrence of the typical symptoms, has been laid out in Table 1.

The following features point to the risk of developing OHSS: young age, low body weight, polycystic ovarian syndrome (PCOS), profound hyperstimulation protocols with gonadotropin-releasing hormone (GnRH) agonist long protocol cotreatment, high number of preovulatory follicles, high serum E2 levels, high (5000 IU) dose of hCG for final oocyte maturation, hCG use for luteal phase supplementation, and a state of pregnancy. Prevention strategies for this condition include cessation of exogenous gonadotropins for several days (referred to as “coasting”), cancellation of the in vitro fertilization (IVF) cycle, and withholding hCG [8,9,10,11]. Moreover, several other preventive interventions can be put in place: follicular aspiration, alternative means of inducing oocyte maturation (e.g., induced endogenous LH surge via a single GnRH agonist bolus dose or short half-life preparation recombinant LH in lieu of hCG), pregnancy prevention during the stimulation cycle through embryo freezing, or the prophylactic infusion of glucocorticoids or albumen [12]. The clinical signs of OHSS are abdominal discomfort, abdominal distention, nausea, vomiting, and diarrhea, and the condition can be managed either on an inpatient or outpatient basis. The complexities inherent in OHSS require absolute compliance with guidelines and evidence-based recommendations, both for the sake of patient welfare and the medicolegal tenability of all procedures. The latter element is especially relevant if an unfavorable outcome arises, which may result in malpractice litigation.

## 2. Materials and Methods

This narrative review draws upon scientific articles found in the PubMed database. The search spanned the 1990–2024 period. Search strings used included “OHSS” or “ovarian hyperstimulation” and “IVF” and “GnRH” and “hCG”. The literature search was limited to studies published in English. The authors included several types of scientific articles: original articles, reviews, case-reports, and evidence-based guidelines and recommendations from international societies and scientific institutions. The initial literature search, which had retrieved 1098 results, was ultimately narrowed down to 111 sources. Only relevant articles were included, i.e., those dealing with the condition’s underlying dynamics, management pathways, and evidence-based criteria and guidelines, which are crucial both from a clinical perspective and from the standpoint of medicolegal tenability as well, for the purpose of diagnosing and treating such a highly consequential condition.

## 3. Results

ART relies on techniques that involve the manipulation of oocytes outside the body [13]. In recent times, there has been a growing utilization of artificial intelligence (AI) in various medical domains, where it combines expertise from different fields, such as computer science, with the help of machine learning algorithms. Currently, there are numerous types of AI technology with various applications in reproductive medicine, such as supervised learning methods and unsupervised learning models. AI itself has the potential to revolutionize the field of reproductive medicine and healthcare as a whole. It can enhance treatment options for patients struggling with infertility, improve procedure planning, and ultimately increase the success rates of ART, leading to cost reductions. By utilizing AI, we can predict clinical outcomes based on initial parameters, eliminating the influence of environmental, emotional, and physical limitations. Although AI will not replace the presence of humans, it will aid in decision-making processes, improving final outcomes and saving time in infertility treatment. However, the integration of AI into ART procedures requires a cautious and thoughtful approach. Legislative and regulatory frameworks must be established, rooted in ethical principles and core values that prioritize human dignity, privacy, data protection, and equality [14].

The first and still the most common ART used is IVF. About 1–4.5% of children are reportedly born from IVF procedures in the USA and Europe [15,16]. Worldwide, more than 7 million children have been born using some form of ART procedure [17]. Although it is possible to perform IVF without medication-induced controlled ovarian hyperstimulation (COH), this natural cycle approach is associated with lower pregnancy rates [18,19]. In most cases, IVF cycles rely on COH to maximize the production of high-quality oocytes [20]. Ovarian stimulation is typically achieved using either exogenous FSH or exogenous human menopausal gonadotropin (hMG) [21,22]. The reproductive axis is regulated by GnRH during IVF using a GnRH antagonist (GnRHant) or GnRH agonist (GnRHa) to prevent premature ovulation [23]. The fundamental purpose is to recruit the highest possible number of mature follicles using gonadotropins and prevent ovulation until the desired follicle count or size is achieved. Once the desired amount or size, along with E2 serum concentration, is reached, a patient is then “triggered” to initiate the ovulatory cascade for final oocyte maturation [24]. Common medications used for triggering include recombinant human chorionic gonadotropin (hCG), GnRHant, or GnRHa [25].

The cumulative live birth rate (CLBR) is a very important index for evaluating the clinical outcomes of IVF/ICSI. The CLBR is influenced by available embryos, number of oocytes retrieved, and age. In order to obtain a higher CLBR and reduce complications, it is necessary to design ovarian stimulation protocols based on the number of target oocytes [26,27].

IVF is aimed at avoiding overstimulation and OHSS, among other things. This condition is characterized by arteriolar vasodilation and increased capillary permeability, causing a shift of the intravascular volume to the extravascular space. Moreover, it leads to significant ovarian enlargement and overproduction of pro-inflammatory and vasoactive cytokines. OHSS onset has been linked to hCG administration as an ovulatory trigger, in light of the ability of hCG to foster vascular endothelial growth factor (VEGF) production, which promotes angiogenesis and increased vascular permeability. The severity of OHSS is directly related to the levels of VEGF. OHSS is also characterized by elevated levels of pro-inflammatory immune cytokines, such as interleukin (IL)-1β, IL-6, IL-8, and tumor necrosis factor α, which further contribute to increased capillary permeability. OHSS clinical manifestations may be ascribed to higher levels of vascular permeability and the ensuing loss of protein-rich fluid to the extravascular space [28,29,30,31,32,33,34,35,36]. Comparing the long protocol with GnRHa, the risk of a severe form of OHSS is reduced by 50% if we use GnRHant. However, a mild or moderate form of OHSS is also possible if the antagonist protocol is used, especially when hCG is used for the final maturation of oocytes in so-called “high responders” patients. At the time of ovulation, triggering GnRHa compared with human chorionic gonadotropin hCG for oocyte triggering and the use of human albumin with a volume expander have been shown to be more effective in reducing OHSS [37]. Induction of ovulation with clomiphene is rarely associated with OHSS but is possible [38,39]. Rare cases of hyperstimulation in a spontaneous cycle are most often associated with high β-hCG levels, multiple pregnancies, and molar pregnancies [40]. Cases of recurrent OHSS have been reported in certain families and are associated with a mutation at the FSH receptor level with less specificity for the ligand and increased sensitivity to β-hCG [41]. Mutations in the gene for the FSH receptor lead to increased sensitivity not only to FSH, but also to a cross-reaction; that is, the increased sensitivity of the FSH receptor to hormones of a structure similar to hCG and TSH. In addition, mutations in the hCG/LH receptor increase sensitivity to normal values and hCG [40,41,42].

After gaining experience and knowledge over the years, it has been found that using the GnRHa trigger with modified luteal phase support yields reproductive outcomes comparable to those achieved with the hCG trigger in fresh cycles. Additionally, the results with frozen embryos derived from the GnRHa trigger are similar to those from the hCG trigger [43]. One significant advantage of using GnRHa trigger is the significant reduction in or complete elimination of OHSS in high responders. Even normal responders can benefit, as the GnRHa trigger allows for an exogenous progesterone-free luteal phase, which may be a more acceptable option for many women. There has been a recent debate on whether to transfer fresh embryos or freeze them for future use [44]. Instead, a tailored transfer and luteal phase policy is advisable. The GnRHa trigger is considered an optimal tool for this purpose, as it allows clinicians to consider the ovarian response to stimulation when deciding whether to proceed with a fresh transfer or carry out a segmented cycle. While long-term results from larger trials comparing fresh and frozen embryo transfer are still unavailable at the time being, most clinicians and patients still prefer fresh transfer whenever possible [45]. One of the innovative ways to lower OHSS risk is in fact embryo freezing. This approach allows for a period of recovery for the ovary after ovarian stimulation and shedding of the endometrial lining, thus creating an opportunity for a “fresh start” for both the patient and the embryo. Since the higher levels of estradiol following IVF have been associated with increased risks of OHSS, it is worth noting that the estradiol levels in fresh cycles are considerably higher than in frozen cycles. As frozen embryos are implanted after a significant period following ovulation induction, the mother’s body would have had ample opportunity to return to its normal hormonal state. Such newfound normalcy is thought to better reproduce the natural conception path associated with a higher likelihood of success [46].

## 4. Discussion

Based on recent evidence, it is safe to perform the GnRHa trigger and fresh transfer in women with 25 or fewer follicles measuring 11 mm or more on the trigger day. For women with a higher number of follicles, the GnRHa trigger and segmentation of the cycle should be recommended. This approach to IVF treatment is safe, effective, and patient-friendly [43].

### 4.1. Weighing All Risk Factors

Physicians should be aware, and women should be informed, that pregnancies complicated by OHSS may incur a higher hypertension and preterm delivery risks in pregnancy. Premature birth and/or hypertension are most often mentioned as potential perinatological complications of severe OHSS, while some authors also observed a higher rate of gestational diabetes [47]. Courbierre et al. found a higher incidence of pre-eclampsia (21.2% versus 9.2%) and preterm birth (36% versus 10.7%) in 40 OHSS pregnancies compared to a control group of 80 IVF pregnancies without OHSS [48]. A larger study by Haas et al. compared the outcomes of pregnancies in 125 patients complicated by severe OHSS with 157 pregnancies achieved by IVF without developing OHSS and found an increased risk of preterm delivery in singleton pregnancies [49]. Since it is very hard to overcome the feelings of grief and loss arising from infertility, such a condition is likely to cause couples undergoing assisted reproductive technology and those not facing ovulation induction and artificial fertilization to be severely affected in their well-being and psychological adjustment capabilities.

OHSS is associated with a higher likelihood of pregnancy and multiple pregnancies, but also higher risks of adverse pregnancy outcomes [50]. One study that investigated risk factors for the occurrence of OHSS showed that such a complication can be expected somewhat more often in younger pregnant women [51].

### 4.2. Ovarian Reserve and OHSS Pathophysiology

Even if demographics may be key elements for risk stratification, markers of ovarian reserve are generally viewed as more reliable predictors of OHSS risk. Before treatment, antimullerian hormone (AMH) levels and antral follicle count (AFC) have been accounted for and monitored as markers of response to ovarian stimulation, with several thresholds apparently pointing to a high OHSS risk. Consistent research findings point to features linked to a robust response to ovarian stimulation as OHSS predisposing factors. This includes baseline traits such as younger age and a PCOS diagnosis, in addition to elevated ovarian reserve markers, including AFC (>24) and AMH levels (>3.4 ng/mL) [52,53].

Strong evidence links OHSS to stimulation-related factors, such as a large amount of mature range follicles at the trigger (>17–19 mm), elevated estradiol at the trigger (>3500–5000 pg/mL), and an increased number of oocytes retrieved (>15–18 mm). There is insufficient evidence that a genetic predisposition may play a role in the propensity for OHSS [54,55].

OHSS pathophysiology has been shown to be closely linked to an increased vascular permeability of the ovarian and peritoneal capillaries, brought about by ovarian VEGF hypersecretion. Treatment relying on a dopamine-receptor agonist such as cabergoline has been hypothesized to potentially cause a reduction in VEGF production, with ensuing OHSS mitigation [56]. In that respect, the administration of dopamine agonists such as cabergoline to reduce the severity and incidence of OHSS has been supported by a remarkable number of research findings [57,58,59]. In order to stave off the harmful effects on follicular growth, final oocyte maturation, fertilization rate, or subsequent clinical outcome due to the untimely blockage of the VEGF system, cabergoline administration should occur immediately after oocyte retrieval [60].

A systematic review assessing 20 RCTs comparing individualized gonadotropin dosing on the basis of ovarian reserve testing (ORT) vs. uniform gonadotropin dosing has concluded that lower gonadotropin dosing on the basis of ORT contributed to a lower incidence of moderate or severe OHSS (OR 0.58, CI 0.34–1.0) with no significant differences in live birth rates [61].

Elevated serum estradiol levels, usually detected over the course of a robust response to ovarian stimulation, are closely linked to a higher risk of developing OHSS in a moderate to severe form. High serum estradiol suppresses the expression of the KISS1 receptor and increases both VEGF and nitric oxide secretion via estrogen receptor modulation [62]. Because of the relationship between estradiol and VEGF secretion, it has been proposed that the administration of an aromatase inhibitor such as letrozole after the administration of the hCG trigger injection will decrease serum estradiol levels and may reduce the incidence of OHSS. As a non-steroidal aromatase inhibitor, Letrozole can block the human aromatase, and inhibit androgens from converting to estrogens, thereby decreasing the E2 level [63]. At first, Letrozole was used in patients with E2-dependent tumors, such as breast cancer, and was used to induce ovulation. In recent years, Letrozole was gradually applied in women with high risk OHSS. Letrozole is known to potentially lower E2 levels, while data are still rather inconclusive as to whether Letrozole can also lower OHSS incidence; that is why Letrozole should not be viewed as the first-line treatment for OHSS prevention.

Though letrozole for OHSS prevention has not yet been officially acknowledged, promising findings seem to support such an approach as an effective treatment option to lower OHSS incidence [64,65,66].

Albumin has a low molecular weight and an average half-life of 20 days. Its binding and transportation properties have been hypothesized to play a role in OHSS prevention. It is important to note that albumin is a blood-derived product and may lead to allergic reactions, anaphylaxis, and the transmission of viral or unidentified diseases. Because albumin increases plasma oncotic pressure, it may counteract the permeability effect of angiotensin II. Albumin may also bind to vasoactive substances, such as factors related to the renin–angiotensin system and VEGF. However, current evidence as to the efficacy of albumin in the prevention of OHSS is still inconclusive [67,68,69,70].

There are no comparative studies addressing the value of thromboprophylaxis in women with severe OHSS. However, the incidence of this complication and its potentially life-threatening nature mean that thromboprophylaxis should be given to women with severe OHSS and those with risk factors such as reduced mobility, obesity or a pre-existing thrombophilia. Antiembolism stockings should be used in patients admitted to hospital with OHSS for whom chemical thromboprophylaxis is contraindicated, as they are likely to have reduced mobility [71].

An endogenous hCG rise related to a fresh transfer cycle, which can potentially worsen late-onset OHSS symptoms and duration, can be prevented through the elective cryopreservation of all embryos and their subsequent transfer in non-stimulated cycles [72,73].

### 4.3. Evidence-Based Therapeutic Options

A study by Kamath et al. has shown a reduction in the required amount of gonadotropin and the incidence of OHSS after the use of clomiphene or letrozole (LE) [74]. Recent findings have demonstrated how in PCOS patients with high anti-Müllerian hormone (AMH) levels, the letrozole co-administration to GnRH-ant protocols results in a lower OHSS incidence than conventional GnRH-ant protocols. LE co-administration may prove highly effective in preventing OHSS, even in women, such as cancer patients, who are at high risk of potentially life-threatening complications [72]. Some studies show that the dosage of letrozole is very important in the effectiveness of OHSS prevention. The higher dose of 7.5 mg per day showed a more significant reduction in the incidence of OHSS than the lower doses of 2.5 or 5 mg [75]. Although it has shown significant success in the prevention of OHSS, letrozole is still not included in official guidelines. It is therefore necessary to carry out additional, more conclusive studies so that it can officially be entered into evidence-based recommendations for OHSS treatment and prevention. One recent study has shown that oral administration of 7.5 mg letrozole daily for 5 consecutive days may be the best option to prevent OHSS in high-risk women [76].

Fouda et al. recently suggested calcium infusion as a novel strategy for the prevention of OHSS, via intravenous calcium gluconate (10%, 10 mL in 200 mL of physiologic saline), to be administered every day for four days, beginning on the day of ovum pickup [77]. The dopamine agonist cabergoline was introduced as a secondary prevention intervention for OHSS in women at high risk of OHSS who are candidates for ART treatment. A 2024 comprehensive review by Baradawan et al. also centered around calcium vs. cabergoline, ultimately concluding that both agents ultimately resulted in similar pregnancy-related outcomes, although calcium infusion could potentially be more effective than cabergoline in reducing the rate of severe OHSS. The need for further research and high-quality trials was stressed, however, before definitive conclusions can be drawn [78]. From a different perspective, the study of Turktekin N. et al. confirmed the safer use of dopamine agonists compared to calcium gluconate [79]. Dopamine agonists seem to reduce the incidence of moderate or severe OHSS in women at high risk of OHSS [80].

PCOS is one of the most common causes of infertility and the reason to undertake ART. Conventional GnRHant protocols have been shown to be safer and more cost-effective for PCOS patients going through IVF/ICSI cycles than the standard long GnRH agonist protocol, with no negative clinical impact on IVF/ICSI outcomes, according to Kadoura et al. [81]. In the study conducted by Ryan et al. prolonged duration of stimulation was a poor predictor of ART success for all couples, with the exception of PCOS patients [82], who are likely to achieve favorable results after early follicular phase GnRH-a [83].

OHSS can be warded off by metformin, through the reduction in VEGF levels, insulin, and E2, on the hCG triggering day [84]. Acetylsalicylic acid combined with glucocorticoids has been found to be able to stave off severe OHSS and raise the amount of successfully acquired oocytes [85]. Recently, Wu D. et al., in their meta-analysis, concluded that calcium, HES (hydroxyethyl starch solution), and cabergoline are safe and effective in preventing moderate-to-severe OHSS [86].

Finally, Yan B, et al. point to a contribution by the transcriptome analysis which revealed several screenings of differentially expressed genes (DEGs) related to OHSS risk factors in the peripheral blood, indicating that these DEGs may be novel players in OHSS development [87].

The increasing infertility problem in the global population has led to growing ART demand, resulting in ever more doubts regarding the health of children born in IVF programs, focusing, for instance, on the risk factors for developing congenital heart diseases (CHDs) [88] and neurodevelopment disorders in newborns born through ART procedures [89].

The main psychological variables involved in the special risk condition of ART were anxiety and depression, leading to the so-called infertility-stress condition. In order to effectively meet such challenges, specific guidelines should be targeted to enhance mental well-being in dealing with infertility and to influence healthier maternal/paternal-infant attachment [90].

When choosing a treatment protocol, one should take into account one that carries a minimal risk of OHSS, so that effectiveness is not compromised or diminished. Women using clomiphene who have failed to conceive are at high risk of OHSS if administered gonadotrophins for ovulation induction. Weight loss can improve ovulatory status.

Moderate OHSS should be monitored on an outpatient basis to prevent any further progression. Treatment includes thromboprophylaxis, fluid balance and pain relief, while non-steroid drugs should be avoided.

There is no one-size-fits-all method for preventing OHSS, but the chances of developing the syndrome can be reduced by individualizing treatment and categorizing women based on their risk of OHSS [91].

**Figure 1:**
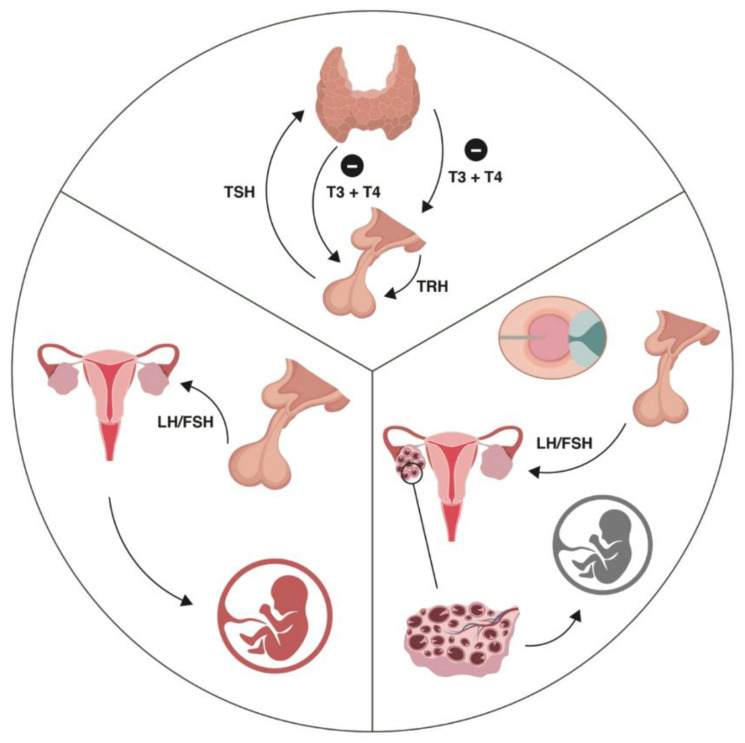
The upper segment represents the negative feedback mechanism between thyroid hormones and TRH/TSH. The right segment (the figure side) presents the gonadotropin effect on folliculogenesis. The left segment (the figure side) presents the impaired hypothalamic–pituitary–gonadal axis, increased LH and decreased FSH, and the increased number of preantral and small antral follicles all contributing to the high serum AMH concentration in PCOS, with the arrest in follicular maturation and its importance for IVF.

A large number of studies have shown that OHSS has a major impact on thyroid function, i.e., on the serum levels of TSH and T4 (Figure 1).

Thus, it is safe to assume that OHSS entails a sudden and supraphysiological increase in serum E2. E2 causes an increased synthesis of TBG, which results in a drop in fT4, and a consequent increase in TSH, via pituitary feedback. Serum TSH also decreases due to the thyrotrophic effect of increasing hCG [92], and the structural similarity of these two hormones is well established [93]. Studies show that the TSH values during COH have a great influence on the reproductive outcome. CPR (clinical pregnancy rate) and LBR have been shown to be impaired in women with elevated serum TSH during COH. High TSH values in COH are also associated with a higher probability of miscarriage, which suggests that higher TSH level during COH could be an indicator of failure to achieve pregnancy later on [94]. The COH-induced elevation in TSH was much higher in patients with basal thyroid diseases. Thyroid function ought to be serially assessed in women with treated hypothyroidism or euthyroid with AITD undergoing OS, beginning from the second hCG measurement for pregnant patients. It is the period after an average of 6 weeks from the start of COH [95].

**Figure 2:**
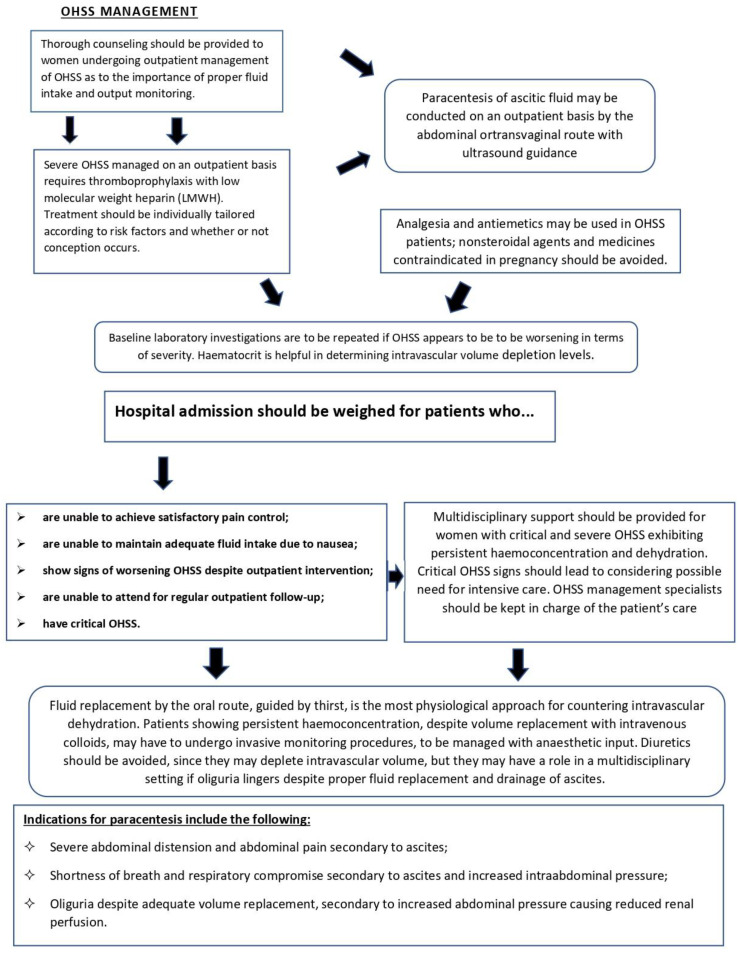
Flowchart outlining evidence-based management pathway for OHSS patients.

Details regarding the acknowledged procedures in the management of OHSS [96,97,98,99,100,101] are briefly outlined in Figure 2.

### 4.4. Guidelines Delineate Therapeutic Pathways for Medicolegal Soundness

According to the 2021 European Thyroid Association Guideline, it is advisable to keep TSH levels in check after COH (in case of pregnancy) in women with AITDs during LT4 treatment, or after the initiation of it. It is not necessary to check the TSH level after COH for euthyroid women without AITD. It is recommended to treat women with proven AITD and with TSH levels > 4.0 mIU/L before COH to keep serum TSH levels < 2.5 mIU/L. It is also suggested to treat women with negative thyroid autoantibodies and with TSH levels > 4.0 mIU/L before COH. It is not recommended to treat euthyroid women without thyroid autoantibodies before COH [95]. There are rare cases of spontaneous ovarian hyperstimulation syndrome occurring in non-pregnant women who have not undergone COH. Ilanchezhian et al. presented a case-report of spontaneous OHSS associated with primary hypothyroidism in a 25-year-old, nonpregnant woman who was not on ovulation induction therapy [97]. This case suggests that in the case of unusual symptoms associated with OHSS, thyroid disorders should be taken into account. Also, there are described cases of severe OHSS in a spontaneously pregnant woman with no acquired diseases [98]. Just as noteworthy are the guidelines issued by the American Society of Reproductive Medicine (ASRM) [99] and the European Society of Human Reproduction and Embryology (ESHRE) [100], which stress how an effort should be made to identify patients at risk for OHSS before stimulation, and how essential it is to select and put in place stimulation protocols aimed at minimizing OHSS risks. A particularly effective strategy is the one relying on the application of GnRH antagonist protocols with a GnRH agonist (with or without low-dose hCG) to induce oocyte maturation. Other approaches that have proven valuable to a degree include the use of cabergoline and cryopreservation of all embryos rather than transfer. If OHSS prevention strategies should prove ineffective, with ensuing severe OHSS, fluid resuscitation, supportive care, paracentesis, and prophylactic anticoagulation are recommended. Similarly, the February 2016 guideline by the Royal College of Obstetricians and Gynecologists [101] points to the need for more conclusive research in order to shed light on the changes in the osmoregulatory system in patients at different phases of OHSS, by relying on well-defined cohorts of women with severe disease who are closely monitored as the condition unfolds. In addition, it is advisable to make comparisons between outpatient and inpatient management of severe OHSS from the standpoints of safety, effectiveness, patient acceptability, and health economic assessment. Such a trial could compare a ‘conventional’ approach of inpatient management, which put in practice conservative indications for abdominal paracentesis, with a more ‘active’ approach which stresses earlier paracentesis on an outpatient basis. Also, more evidence is needed in order to fully assess the role of GnRH antagonists and dopamine agonists in the management of patients diagnosed with established OHSS [99,100,101].

Such resources must be viewed as a valuable means to provide guidance and facilitate timely and appropriate diagnosis for patients at risk for OHSS. The importance and essential nature of guidelines is indisputable in the diagnosis and treatment of OHSS even from a medicolegal standpoint [102]. Compliance with international accredited guidelines and evidence-based recommendations is in fact essential in order to shield healthcare professionals from negligence-based malpractice lawsuits in case of adverse outcomes. Unequivocal evidence-based standards are meant to ensure equal access and care for any patient in need, even under circumstances of absolute emergency such as the COVID-19 pandemic, when healthcare services (including fertility procedures) were negatively affected, despite the virus’ harmful effects on fertility [103,104]. It is therefore essential to frame and apply criteria designed to meet ordinary as well as extraordinary scenarios (e.g., through the use of telemedicine approaches whenever possible, to allay pressure on healthcare services) [105,106]. Objective application and implementation of scientifically acknowledged criteria is essential in terms of medicolegal viability. Particularly under tort law statutes in many jurisdictions, the onus is on doctors and healthcare facilities to prove adherence to guidelines and best practices, hence documentable compliance is of utmost importance, even more so in obstetrics and gynecology, specialties among the ones most at risk of malpractice charges [107,108,109].

It is important to emphasize that a thyroid gland functional disorder occurs very often in OHSS, whether or not there is an associated thyroid pathology, which is why it is imperative to take into account an adequate L4 substitution in order to improve the outcome of pregnancy achieved with the help of ART [110]. Timely screening of the serum TSH level in risk groups is of crucial importance for deciding which pregnant women are candidates for levothyroxine substitution. It is therefore worth bearing in mind that thyroid hormones have a strong influence on all cells in the body, so thyroid disorders can often be misdiagnosed and mistaken with other conditions, including OHSS [111].

## 5. Conclusions

Undergoing ART programs to restore fertility is becoming increasingly widespread, leading to ever-higher numbers of patients being admitted to emergency departments with complications. OHSS is one of the most frequent complications, accounting for 30% of IVF cycles. Young age, low body mass index, PCOS, previous OHSS, high follicle count, and elevated serum E2 are certain factors that may predispose women to OHSS. In addition to OHSS, patients may also experience complications such as infection, thromboembolism, acute respiratory distress syndrome, acute coronary syndrome, and severe shock. Providing effective care for OHSS patients begins with early diagnosis, while also evaluating for comorbidities and complications. In addition to that, we should pay more attention to the psychological component of this complication and of infertility as a whole.

## Figures and Tables

**Table 1:** OHSS classification.

Symptoms Frequency	Always	Usually	Often	Rarely
Mild OHSS	Enlargement of bilateral ovaries with multiple follicular.	Corpus luteal cysts, measuring up to 8 cm.	Abdominal bloating and mild abdominal pain.	
Moderate OHSS	Ovaries up to 12 cm.	Abdominal bloating due to an increase in ovarian size.	Gastrointestinal symptoms (nausea, vomiting and diarrhea).	Ultrasound evidence of ascites and rapid weight gain of over 2.5–3 kg.
Severe OHSS	Large ovarian cysts (>12 × 12 cm), clinical ascites with or without hydrothorax.	Hyperkalemia (potassium > 5 mmol/L), hyponatremia (sodium < 135 mmol/L).	Oliguria (<300 mL/d or <30 mL/h), creatinine 1.1–1.5 mg/dL, and hypovolemic shock. Hemoconcentration with hematocrit > 45%, white cell count > 15,000.	Liver dysfunction, increased blood viscosity, and thromboembolic.
Critical OHSS	Severe ascites or hydrothorax, hematocrit > 55%, white cell count > 25,000/mL.	Oliguria or anuria, creatinine ≥ 1.6 mg/dL, creatinine clearance < 50 mL/min.	Thromboembolism, or acute respiratory distress syndrome.	

## Data Availability

Data are available upon request from the corresponding author.
